# Periosteal Osteosarcoma: A Single-Institutional Study of Factors Related to Oncologic Outcomes

**DOI:** 10.1155/2018/8631237

**Published:** 2018-09-27

**Authors:** Chung Ming Chan, Adam D. Lindsay, Andre R. V. Spiguel, C. Parker Gibbs, Mark T. Scarborough

**Affiliations:** ^1^Division of Orthopaedic Oncology, Department of Orthopaedics and Rehabilitation, University of Florida, Gainesville, FL, USA; ^2^Department of Orthopaedic Surgery, University of Connecticut, Farmington, CT, USA

## Abstract

**Background:**

Periosteal osteosarcoma is a rare surface-based variant with a lower propensity to metastasis and better prognosis than conventional osteosarcoma. The literature supporting survival benefit with adjuvant chemotherapy is lacking. Our institutional practice is for chemotherapy to be offered to patients with high-grade disease.

**Methods:**

We conducted a retrospective cohort study of patients managed for periosteal osteosarcoma from 1970 to 2015 analyzing the survival outcomes and assessing for any relationship of survival to patient- or treatment-related factors. 18 patients were included. The study population presented at a mean of 20.8 years and was followed for a mean of 10.7 years. Factors assessed for an association with survival included age, size of tumor, use of chemotherapy, presence of medullary involvement, presence of high-grade disease, local recurrence, and site of disease. Kaplan–Meier survival analysis and Cox proportional hazard regression were performed to calculate the survival rates and to assess for the effect of any factor on survival.

**Results:**

10-year overall survival rate was 77.1%, and 10-year event-free survival rate was 66.4%. No factor was found to have an association with overall or event-free survival.

**Conclusion:**

These findings add to the available evidence which has failed to find any survival benefit from chemotherapy; patients with this rare disease and their families should be counselled regarding the unclear role of chemotherapy in this rare subtype of osteosarcoma.

## 1. Introduction

Periosteal osteosarcoma is a rare intermediate-grade malignancy, estimated to represent less than 2% of all osteosarcomas. It is characterized by its histologic appearance of being predominantly chondroblastic with areas of osteoid and its radiographic appearance of a periosteally based lesion with periosteal reaction projecting perpendicularly into the associated soft tissue and has been found to have a lower propensity for metastasis compared to conventional, high-grade medullary osteosarcoma [1–7].

The difference in the natural history of periosteal osteosarcoma as compared to conventional intramedullary osteosarcoma had been observed by the early authors on this topic such as Lichtenstein [6]. This unique and rare subtype of osteosarcoma was further delineated by authors such as Unni et al. [8] and Campanacci and Giunti [4] where the authors made the case for this being recognized as a distinct entity on the grounds of the unique radiographic and histologic features with a clinical behavior distinct from other surface-based osteosarcomas such as parosteal osteosarcoma [9] and high-grade surface osteosarcoma [10]. The importance of making the diagnosis of periosteal osteosarcoma cannot be understated owing to the distinct natural history and that resection alone is a standard treatment for parosteal osteosarcoma [9], while resection and adjuvant chemotherapy are standard treatments for high-grade surface osteosarcoma [10].

While wide resection for local control of this malignancy is accepted as the cornerstone of treatment, strategies differ as to whether adjuvant chemotherapy is administered in patients with this diagnosis. Recent large studies have been inconclusive regarding the utility of adjuvant chemotherapy, with no clear association of the use adjuvant chemotherapy with increased survival [11, 12].

The purpose of this study was to describe our institution's experience of management of patients with this rare malignancy with respect to the characteristics of this patient group and their survival outcomes. We also sought to assess if any patient- or treatment-related factors were associated with improved survival.

## 2. Materials and Methods

This study is a retrospective case series. The prospectively collected institutional musculoskeletal oncology database was queried to identify patients who had been diagnosed with and managed for periosteal osteosarcoma. We identified 19 patients who had been managed during the period from 1970 to 2015. Of the 19 patients, one had less than one year follow-up and was excluded from statistical analysis. The study population of 18 patients comprised 6 males and 12 females who presented at a mean age of 20.8 years (±9.8) and were followed for a mean of 10.7 years (±7.4).

Data were collected from the patient clinical and pathological charts on demographic characteristics of subjects, tumor-related features, the nature of surgical and medical treatment, and the occurrence of any significant events related to the malignancy (i.e., local recurrence, metastasis, development of a second malignancy, and death). Where patients were histologically graded on the four-point Broder's scale, grades I and II were classified as low grade and grades III and IV were classified as high grade. Patient data regarding certain tumor-related and treatment-related factors were collected for analysis. The factors assessed included the age at diagnosis, size of tumor (as assessed by the maximum dimension measured in centimeters), the presence of high-grade tumor, location of the tumor (axial location versus appendicular), the presence of medullary extension or invasion, and whether chemotherapy was administered. The maximum tumor dimension was recorded for 10 of 18 of our study population. Patients were followed up in the clinic and had radiographic evaluations of the site of disease to monitor for local recurrence as well as of the chest for pulmonary metastasis. Owing to the subjects having been managed from 1970 to 2015, the standard surveillance protocol for pulmonary involvement involved chest radiographs for subjects managed earlier and computed tomography of the lungs for the first 5 years for patients managed more recently. The interval for surveillance was 3 months for the first two years, 4 months for the third-year postsurgery, 6 months for the fourth- and fifth-years postsurgery, and yearly thereafter. Where the death of a study subject could be ascertained, this was included for analysis of overall survival.

Descriptive statistical analysis and Kaplan–Meier survival analysis were performed for the whole study group. Both overall survival and event-free survival were analyzed. Events were defined as local recurrence, metastasis, or mortality. The presence of any association between survival and patient- and tumor-related factors was assessed using the log-rank test and Cox proportional-hazards regression. Patient subgroups were compared and analyzed using a Cox proportional-hazards regression model which was used for analysis to assess for the presence of any association with survival outcome.

Statistical analysis was performed using EZR, a statistical software package based on R (Easy R, Version 2.13.0; Jichi Medical University, Saitama, Japan) [13]. Significance was determined using a 95% confidence level.

## 3. Results and Discussion

### 3.1. Results

**Figure 1:**
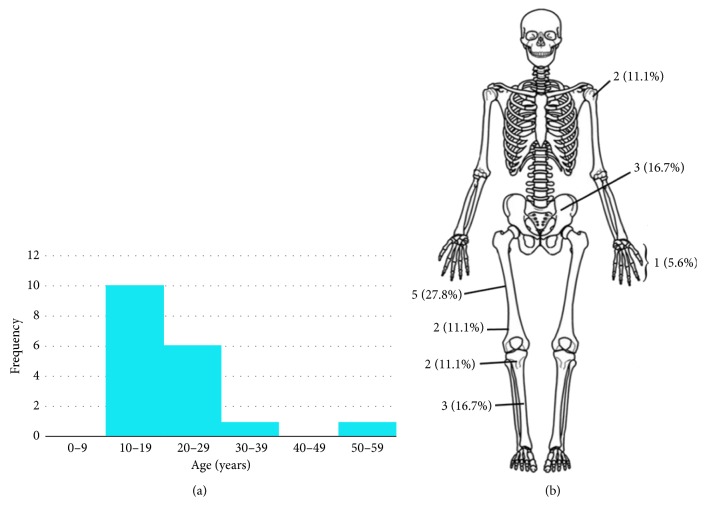
Distribution of primary tumors in study population by age and location.

The commonest site of disease was the femoral diaphysis (5), followed by the tibial diaphysis (3) and the ilium (3) (Figure 1). 12 of 18 patients had tumors that were of high grade, and 11 of these 12 patients received chemotherapy. 17 of 18 patients underwent wide resection of the primary tumor for local control, while the last patient's resection was a marginal resection. The surgery performed was limb sparing in 14 of the 18 patients with an overall 78% limb salvage rate. Medullary involvement was noted in 9 of 18 patients.

One patient (5.3%) developed a local recurrence 9 months after an intralesional excision was performed at the referring institution. This recurrence was not associated with dedifferentiation. Two patients (10.5%) developed metastasis while on follow-up. The first patient developed an isolated lung metastasis at 19 months. This was managed with chemotherapy and pulmonary metastasectomy. There was no evidence of disease for up to 75 months after the diagnosis of the metastasis. The second patient developed a proximal humeral metastasis at 94 months; this was treated with chemotherapy and resection of the proximal humeral metastasis. The patient subsequently developed pulmonary metastases and eventually succumbed to the disease 16 months after the diagnosis of the proximal humeral metastasis.

**Figure 2:**
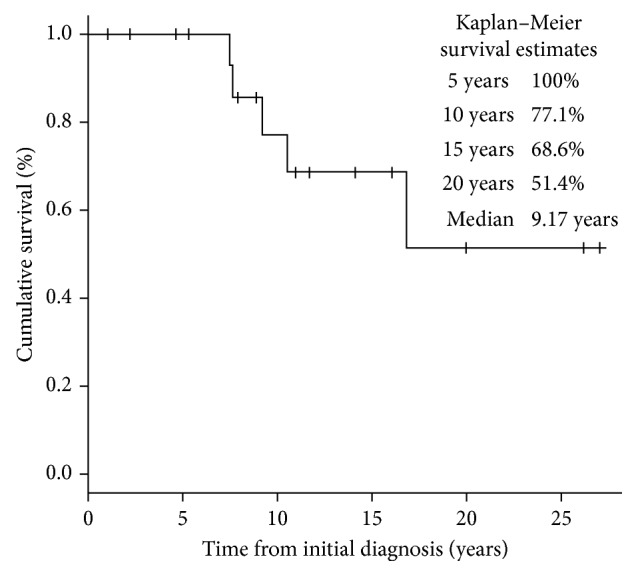
Kaplan–Meier curve of overall survival of study population.

**Figure 3:**
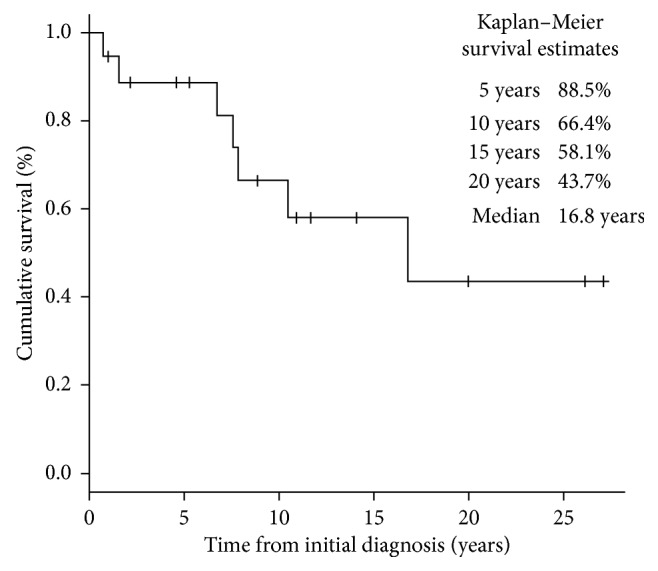
Kaplan–Meier curve of event-free survival of study population.

**Figure 4:**
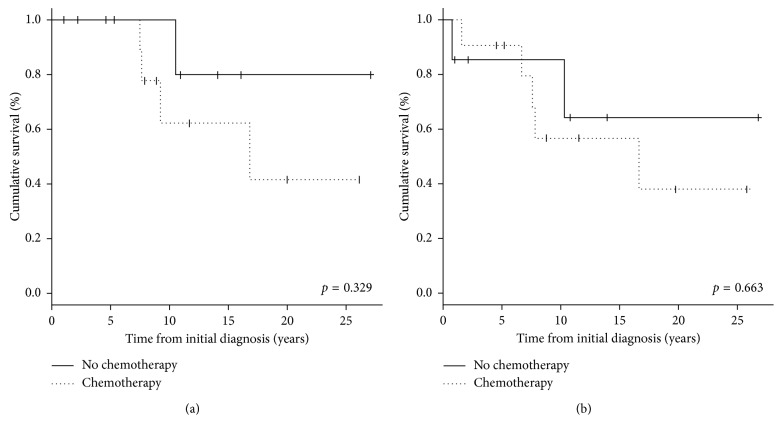
Kaplan–Meier curves comparing overall survival (a) and event-free survival (b) of patients who did and did not receive chemotherapy.

The 10-year overall survival rate was 77.1% (Figure 2) and 10-year event-free survival rate was 66.4% (Figure 3). No factor was found to have a significant association with survival (Table 1). Notably, no survival benefit was noted with the use of chemotherapy (Figure 4). Multivariate analysis was not performed as no factor was found to have a significant association with survival.

### 3.2. Discussion

Survival in patients with periosteal osteosarcoma is relatively high compared to other commoner subtypes of osteosarcoma, and the use of adjuvant chemotherapy has not been found in several studies to be associated with increased survival. The 10-year survival rate of 77.1% in this study is comparable to that in the literature [2, 3, 11, 12, 14, 15] (Table 2).

In this study, we did not exclude patients with the presence of medullary involvement. Studies such as by Unni et al. [8] and Rose et al. [2] have excluded patients with medullary involvement from their studies as it is argued that it is not possible to definitively distinguish the tumors in those cases from classic chondroblastic osteosarcoma. Many other contemporary studies [11, 12, 14, 15] have abided by the plea for acceptance of medullary involvement in diagnoses of periosteal osteosarcoma by Hall et al. [16], as this is seen as the natural progression of the disease. This is the practice at our center as well (Table 2).

There is a wide variation in the rates of high-grade tumors in the studies on periosteal osteosarcoma in the literature. In our study, 12 of 18 patients (66%) were classified as having high-grade tumors or tumors with foci of high-grade tumor. Of these 12 patients, 2 were initially graded as low grade on the biopsy specimens and were subsequently reclassified as high grade following complete tumor resection and pathological examination of the entire tumor. In the study by Revell et al. [14], all patients were classified as intermediate or high grade; this was the case as well in the study by Rose et al. [2], with 11 of 29 subjects having intermediate-grade tumors and 18 of 29 subjects having high-grade tumors. Other studies have significantly higher rates of high-grade tumors. The study by Cesari et al. [11] actually reported a 94% rate of tumors being grade 3/high grade, and in that study, 14 patients received chemotherapy. In that study, the pathology was reviewed specifically for the study and 31 of 33 patients were classified as high grade and it is unclear if this could be accounted for by the areas available for repeat review being areas of higher grade involvement. In the study by Grimer et al. [12], 44 of the 51 patients (86.2%) where the information was available for review had high-grade tumors. In their discussion of the issue of tumor grade, the possibility of a high-grade surface osteosarcomas being misclassified as periosteal osteosarcoma was raised in their discussion owing to the multi-institutional nature of the study and as there was no centralized review of pathology. The importance of correct classification of the tumor grade cannot be overstated as this is often a criterion in deciding on which patients to receive chemotherapy.

Regarding adjuvant chemotherapy, the 12 patients with high-grade tumors were recommended to receive chemotherapy, and all but one of those 12 received chemotherapy. The overall and event-free survival of patients with high-grade tumors was generally poorer than those without high-grade tumor; similarly, the survival was poorer in those patients who received chemotherapy than in those who did not receive chemotherapy. This difference however was not found to be statistically significant on univariate analysis with the log-rank test. Multivariate testing was noted performed owing to the lack of any factor showing statistical significant differences. These findings of there being no significant difference in survival between patients who did and did not receive chemotherapy echo the findings of the multicenter study by Grimer et al. [12] involving 119 subjects and the largest single-institution study by Cesari et al. [11] involving 33 subjects.

Our practice is to recommend neoadjuvant chemotherapy to patients with high-grade tumors on initial biopsy and adjuvant chemotherapy to patients where the resected tumor reveals foci of high-grade disease not found on the initial biopsy. In our study, five of the 12 patients who received chemotherapy received it prior to surgery. The pathology reports for four of these five patients featured estimates of tumor necrosis, and they were <50%, 90%, 92%, and >90%. This may be seen as suggestive that chemotherapy is active against periosteal osteosarcoma. Of particular note, two of these four patients had tumor without evidence of medullary involvement which fits the more restrictive case definition of periosteal osteosarcoma applied in some studies. The tumor necrosis was reported as 90% and >90% in these two patients. These findings are in contrast to those of the study by Rose et al. [2] where tumor necrosis in the two of 29 patients who received neoadjuvant chemotherapy was 10–20%. While the study by Revell et al. [14] did not include a discussion of histologic response to chemotherapy, the 100% 10-year survival rate observed was attributed to the radical tumor resection and the use of chemotherapy.

The survival curves in this study show persistent reduction in the survival estimates with time suggestive of the development of late events with the event-free survival (EFS) dropping from 66% at 10 years to 58% at 15 years and then to 44% at 20 years. This contrasts to the study by Rose et al. [2] where all local and distant recurrences were noted within 36 months and the disease-specific survival curves plateaued, with that data suggesting a low risk of late clinical events. Several explanations could account for these differences: firstly, in our study, events included not only local recurrence and metastasis but also second malignancies and death from all causes. A similar definition for events was used in the series by Cesari et al. [11] where a similar EFS at 10 years of 52% was noted. These events of second malignancy and death from other causes may not be accounted for in some other studies where the disease-specific survival curves plateau. Another significant factor that may account for these differences is the impact of censoring in the calculation of the Kaplan–Meier survival estimates in our small series where at 10 years, 39% of subjects had been censored, while at 15 years, it was 50%. Consider an assumption of censoring in survival analysis is that the subjects censored have a similar prospect of survival as those that continued to be followed. In our study, where patients were found to have died even after their last clinical follow-up, their death was included as an event for survival analysis. If the patients who were lost to follow-up were at a lower chance of events and death, this could have biased the analysis to estimating a poorer rate of survival.

The risk of second malignancy is well known in patients treated for childhood malignancies and in osteosarcoma as well [17]. The benefits of the use of chemotherapy in many childhood cancers outweigh the relatively small risk of a second malignancy, but should be raised as a concern in discussing the use of chemotherapy in patients with periosteal osteosarcoma where its utility has not been well proven. In the study by Ritts et al. [5], of their 22 patients, 3 developed another malignancy, 2 developed acute myelocytic leukemia (AML), and 1 developed liposarcoma, whereas one patient in the study by Revell et al. [14] developed AML as well. In our study, one patient was diagnosed with mesenchymal chondrosarcoma at the same site of her periosteal osteosarcoma 81 months later, and died 8 months after that diagnosis. In the series by Cesari et al. [11], similarly, one patient died from mesenchymal chondrosarcoma at 128 months after the diagnosis of periosteal osteosarcoma, but at a site different from the primary periosteal osteosarcoma. Other second malignancies occurring in that study were breast cancer and acute lymphoblastic leukemia.

Our study has several limitations. It is a small series and it spans 5 decades where some variation in chemotherapy protocols existed. This study also only reports overall survival, as we were not able to ascertain the cause of death on all subjects who died and thus could not report disease-specific survival.

Making strong recommendations regarding the utility of adjuvant chemotherapy of periosteal osteosarcoma is challenging owing to its rarity and the limited and varied data. Owing to the rarity of this disease, it is unlikely that a randomized study will be conducted to answer this question and a large multicenter retrospective study [12] of this rare disease has been unable to provide clear evidence of a survival benefit with the use chemotherapy in this patient group. As in the management of other subtypes of osteosarcoma, wide resection remains the preferred mode of surgical treatment. Similar to other studies on this disease, this study does not conclusively support or refute the use of chemotherapy. The presence of tumor necrosis in four of the six patients in our study who received neoadjuvant chemotherapy could be seen to suggest activity of chemotherapy against periosteal osteosarcoma and lend support to the practice of offering chemotherapy to patients with high-grade disease.

The silence of the literature regarding the efficacy of chemotherapy on periosteal osteosarcoma will lead clinicians to draw on their experience of other subtypes of osteosarcoma, in particular that of high-grade conventional osteosarcoma, to make treatment recommendations. It should be emphasized to patients and their families that little can be said definitively owing to the rarity of the condition, but that current studies have not proven the efficacy of chemotherapy in improving survival outcomes in patients with periosteal osteosarcoma.

## Figures and Tables

**Table 1:** Factors assessed for the effect on survival.

	Overall survival			Event-free survival		
	HR	CI	*p* value	HR	CI	*p* value
Age (continuous variable)	0.920	0.72–1.175	0.5025	0.9131	0.758–1.10	0.340
Size (continuous variable)	0.864	0.5515–1.354	0.5243	0.738	0.513–1.06	0.103
Use of chemotherapy	2.90	0.3125–26.83	0.3493	1.443	0.275–7.563	0.664
Medullary involvement	1.01	0.1637–6.189	0.9944	2.151	0.411–11.27	0.365
Presence of high-grade disease	1.00 × 10^9^	0–∞	0.9992	2.278	0.272–19.1	0.448
Local recurrence	2.785	0.286–27.13	0.3778	—	—	—
Site (axial versus appendicular)	2.699	0.4407–16.52	0.2826	1.415	0.269–7.44	0.682

Hazard ratios (HRs) with confidence interval (CI) and *p* values calculated using the Cox proportional-hazards method with each factor being assessed individually.

**Table 2:** Summary of the contemporary literature regarding periosteal osteosarcoma.

Study	*N*	Gender ratio (M : F)	Average age of presentation (range) (years)	Mean follow-up (range) (years)	10-year overall survival (%)	Chemotherapy used (%)	Medullary involvement (%)	Local recurrence (%)	Metastasis rate (%)
Bertoni et al. [3]	20	9 : 11	Mean 19.6 (11–53)	7.9 (0.4–39)	85	10	—	40	15
Hall et al. [16]	6	3 : 3	Mean 25.3 (15–40)	2.9 (1.1–5)	—	50	50	0	16.7
Ritts et al. [5]	22	8 : 14	Mean 20.5 (9–47)	10.0 (1.4–29.3)	71.3	9.1	0	13.6	13.6
Revell et al. [14]	17	10 : 7	Median 18 (10–35)	6.8 (0.8–16.7)	88	82	23.5	5.9	0
Grimer et al. [12]	119	54 : 64	Median 18 (8–72)	7.1 (0.5–21)	83	68	—	6.7	14.2
Rose et al. [2]	29	13 : 16	Mean 20.6 (9–47)	15.8 (4–51)^*∗*^	83	31	0	17.2	17.2
Cesari et al. [11]	33	19 : 14	Median 16 (6–32)	Median 10.1 (0.75–30.5)	84	42	65.2^†^	21.2	12.1
Gulia et al. [15]	18	12 : 6	Mean 16.3 (5–26)	Median 5.1 (1.5–10.8)	83.3 (5 y OS)	89	44.4	11	22.2
This study	18	6 : 12	Mean 20.8 (10–56)	10.4 (1–27.1)	77.1	61	50	5.6	11.1

^*∗*^Surviving cohort; ^†^23 of 33 subjects assessed had medullary involvement.

## Data Availability

The data that support the findings of this study are available from the corresponding author, CMC, upon request.
